# Access to mental health services: the perspective of people experiencing homelessness

**DOI:** 10.1590/0034-7167-2025-0045

**Published:** 2026-07-24

**Authors:** Tatiane Cristina da Silva, Aldair Weber, Giulia Delfini, Vanessa Pellegrino Toledo

**Affiliations:** I Unversidade Estadual de Campinas. Campinas, São Paulo, Brazil

**Keywords:** Homeless Persons, Mental Health, Mental Health Services, Effective Access to Health Services, Delivery of Health Care., Personas en Situación de Calle, Salud Mental, Servicios de Salud Mental, Acceso Efectivo a los Servicios de Salud, Atención a la Salud.

## Abstract

**Objectives:**

to understand the access of people experiencing homelessness to mental health services within the Psychosocial Care Network in a municipality in the interior of São Paulo State.

**Methods:**

a qualitative study based on Alfred Schutz’s phenomenological sociology, involving interviews with 21 individuals experiencing homelessness conducted in July 2024. The analysis revealed two categories: “Experiences of people experiencing homelessness in accessing the Psychosocial Care Network” and “Expectations in light of lived experiences”.

**Results:**

mobility between points of care within the network is driven by barriers in mental health care, while the presence of the Street Outreach Clinic facilitates access. Both continuity and discontinuity in care were observed, linked to experiences of welcoming as well as discrimination. Expectations include equal and universal access to care.

**Final Considerations:**

access, marked by vulnerability, inadequate support, discrimination, and tension due to care disruption, demands welcoming practices, improved service quality, and professional training.

## RESUMEN

**Objetivos:**

comprender el acceso de la población en situación de calle a los servicios de salud mental de la Red de Atención Psicosocial en un municipio del interior del estado de São Paulo.

**Métodos:**

estudio cualitativo basado en la sociología fenomenológica de Alfred Schutz, con entrevistas realizadas a 21 personas en situación de calle en julio de 2024. El análisis reveló dos categorías: “Experiencia de la población en situación de calle en el acceso a la Red de Atención Psicosocial” y “Expectativas frente a las experiencias vividas”.

**Resultados:**

la movilidad entre los puntos de atención de la red está motivada por barreras en el cuidado de la salud mental, y la presencia del Consultorio en la Calle facilita el acceso. Se observa tanto continuidad como interrupción en el cuidado, asociadas a experiencias de acogida y también de discriminación. Las expectativas incluyen una atención igualitaria y universal.

**Consideraciones Finales:**

el acceso, marcado por la vulnerabilidad, el apoyo insuficiente, la discriminación y la tensión provocada por la ruptura del cuidado, requiere prácticas de acogida, mejora en la calidad de la atención y capacitación profesional.

## INTRODUCTION

Globally, it is estimated that 150 million people are experiencing homelessness^(1)^. In Brazil, according to data from the “*Cadastro Único para Programas Sociais”* (“*CadÚnico”,* Unified Registry for Social Programs), approximately 221,000 individuals were registered between 2018 and July 2023^(2,3)^. Campinas, a city in the interior of São Paulo State, ranks among the ten municipalities that concentrate 48% of the People Experiencing Homelessness (PEH) in the country, recording 1,905 individuals in 2021 and 2,547 in 2023, also according to *CadÚnico* ^2^. However, data from the Municipal Department of Social Assistance and Development indicated 932 people living on the streets in 2021 and 1,300 in 2024, revealing discrepancies in the representation of this population’s reality^(4,5)^.

Internationally, homelessness is considered a consequence of structural inequalities, poverty, social exclusion, and the absence of integrated policies^(6)^. In Brazil, the “*Política Nacional para a População em Situação de Rua”* (“*PNPSR”*, National Policy for the Homeless Population)^(7)^, established in 2009, defines PEH as heterogeneous and socially vulnerable, sharing experiences of extreme poverty, weakened family and emotional ties, and the absence of regular conventional housing^(7)^. In this context, vulnerability is understood as a unique and complex phenomenon resulting from the interaction of individual, social, and programmatic dimensions. This concept is used to understand the susceptibilities that expose individuals to health problems and risks^(8)^. Homelessness, in turn, is a result of the prevailing mode of production, exclusion from the labor market, unplanned urbanization, cyclical economic crises, and deep social inequality^(9)^.

In this context, PEH use public spaces, shelter units, and degraded or abandoned areas as permanent or temporary places of refuge, seeking means of subsistence within these environments^(10)^. The phenomenon of living on the streets emerges from deep social inequalities and is manifested through factors such as unemployment, deteriorating living conditions, substance abuse (alcohol and other drugs), broken family ties, migration, reentry after incarceration, threats to safety in places of origin, personal decisions, and physical and mental health issues^(9,10)^.

PEH face daily vulnerability due to the lack of safe shelters and difficulties accessing food, hygiene, rest, and essential health care, exposing them to constant uncertainty and extreme precariousness^(9,10)^. This context heightens their exposure to risk factors and contributes to the onset and worsening of mental health disorders^(11)^. These conditions are associated with socioeconomic factors, fragile support networks, low educational attainment, unemployment, and weakened social ties, resulting in high rates of depression, bipolar disorder, schizophrenia, and substance dependence^(11,12)^. The overlap between street life and psychological suffering increases social exclusion, hinders access to health services, and compromises continuity of care, further intensifying the vulnerability of this population^(9-12)^.

In light of this, public policies in Brazil, such as the *“PNPSR”* ^(7)^, aim to ensure comprehensive care, strengthen actions within the “*Sistema Único de Saúde”*, (*“SUS”*, Unified Health System), and guarantee broad, community-based, and safe access to health services, including the “*Rede de Atenção Psicossocial”* (*“RAPS”,* Psychosocial Care Network)^(7)^. However, the principles of universality and equity that underpin *“SUS”* remain distant from the reality of the PEH, whose access to health care is irregular and generally driven by emergencies that threaten their survival^(13)^. In such cases, care is most often sought at Emergency Care Units (*“UPA”*), to the detriment of primary care services that focus on prevention and health promotion^(13,14)^.

In this scenario, access to specialized psychosocial care services, such as the “*Consultório na Rua”* (“*CnaR”*, Street Outreach Clinic), is limited for the PEH, depending on matrix support and the mediation of social assistance institutions^(15)^. Furthermore, the territorial mobility of part of PEH population poses a challenge to the organization of health services based on geographic boundaries and assigned populations, affecting the planning of actions and continuous follow-up^(5,14)^. This dynamic results in fragmented and discontinuous care, which negatively impacts mental health by weakening therapeutic bonds, trust networks, and welcoming practices^(5,14)^.

In this context, the vulnerability and continuous growth of PEH demand the production and systematization of consistent official data on the lived reality of this group, in order to support the formulation of effective public policies aligned with the principles of the *“PNPSR”*. The lack of accurate information compromises the true measurement of this population and hinders the planning of intersectoral actions^(3-5)^.

Therefore, this study is justified by the demographic scenario and the lived context of PEH, in which sporadic and discontinuous access to health services makes it difficult to plan effective actions for this population^(7,13-15)^. These barriers affect the mental health of PEH, undermining the development of therapeutic bonds and trust networks that are essential for comprehensive care^(13-15)^. These aspects reinforce the need for studies that deepen the understanding of the dynamics of access and care for PEH within health services, creating space for the expression of the phenomenon from the perspective of those who experience it.

## OBJECTIVES

To understand the access of PEH to mental health services within the *“RAPS”* in a municipality in the interior of São Paulo State.

## METHODS

### Ethical aspects

The study was approved by the Research Ethics Committee of the State University of Campinas in 2024 and followed the ethical guidelines established by Resolution No. 466/12 of the “*Conselho Nacional de Saúde”* (Brazilian National Health Council). Participants signed the Free and Informed Consent Form, agreed to audio recording, and were identified by the letter “P” followed by Arabic numerals, ensuring confidentiality and chronological order of the interviews.

### Theoretical and methodological framework

Alfred Schütz proposes the understanding of the reasons behind past experiences (“reasons-why”) and the intentions directed toward future actions (“reasons-for”), which drive and project individuals into their lifeworld^(16)^. This lifeworld is understood as an intersubjective setting inhabited by human beings, where they carry out actions, establish interactions, and assign meanings through face-to-face relationships and biographical aspects that together form their stock of knowledge^(16)^. Among the various typifications found in phenomenological sociology, the lived type refers to actions within the social world, interpreted and organized based on motivations^(16)^. From these experiences, common patterns of meaning are constructed, helping individuals to understand and interpret shared reality^(16)^.

### Type of study

This is a qualitative study guided by the Consolidated Criteria for Reporting Qualitative Research - COREQ^(17)^ and supported by the theoretical and methodological framework of Alfred Schütz^(16)^. This approach enables a deep understanding of the access of PEH to mental health services, as it reveals the essence of the phenomenon by considering the street as their lifeworld, an environment shaped by lived experiences, motivations, biographical aspects, stock of knowledge, intersubjective experiences of everyday life, and the meanings attributed to their actions^(16)^.

### Study setting

The study was conducted in a municipality in the interior of São Paulo State with approximately 1.1 million inhabitants, featuring developed urban infrastructure, reference institutions, and industrial, technological, and service hubs^(18)^. In this setting, the city records a high rate of PEH, despite contradictions in the data regarding individuals living on its streets^(2,4)^.

Participants in this study were individuals experiencing homelessness assisted by the municipality’s *“CnaR”*, a point of care within the *“RAPS”* that provides mobile services aimed at supporting this population^(19)^. Led by a multidisciplinary team, the *“CnaR”* carries out health promotion and prevention activities tailored to the needs of PEH, working in an integrated manner with primary care units, specialized services, and territorial health surveillance teams^(20)^. The three *“CnaR”* teams perform approximately 800 consultations per month^(20)^. However, this study focused on only one team, responsible for activities in the city’s central region, strategically located near the highest concentration of PEH. The team provides care through mobile units at two fixed locations in the area, aiming to establish them as reference points for PEH to access the service.

### Data sources

Inclusion criteria were being at least eighteen years old, experiencing homelessness, and receiving care from the *“CnaR”*, a mental health service within the *“RAPS”*, at the time of data collection. The length of time spent living on the streets was not used as a criterion, as the phenomenon was understood through the subjective aspects of the PEH’s experience, regardless of when it occurred^(16)^. The exclusion criterion was individuals who had difficulty verbalizing due to medication effects or psychological disorder.

### Methodological procedures, data collection and organization

The researchers met with the coordination team of the *“CnaR”* to align the start of data collection, which was conducted in June and July 2024 by the first author, a nursing undergraduate at the time, under the supervision of the research team to ensure proper execution. In addition to prior experience working with PEH, the researcher also conducted pilot interviews, which were transcribed and analyzed to refine the data collection technique. During this period, the researcher introduced herself to the “*CnaR”* team and attended the service regularly to become familiar with its workflow and establish initial contact with the PEH. Following this phase, data collection began at the fixed locations where *“CnaR”* activities were carried out. Individuals who met the inclusion criteria were invited to participate in the study, and the researcher explained its purpose to them. The *“CnaR”* team remained present during the interviews but did not interfere. Convenience sampling was used, and interviews were conducted at two fixed locations - public squares where the service provided outdoor tables and chairs near the mobile unit - with the aim of creating a welcoming space for the PEH.

Phenomenological, individual, non-directive, and semi-structured interviews were conducted^(21)^, guided by the central question: “How did you access mental health services?”. From this, additional questions emerged that allowed for deeper exploration and understanding of the phenomenon under study, such as: “Tell me about your experience attending mental health services” and “How did you expect that care to be?”. During the interviews, the researcher’s assumptions were set aside through the adoption of a phenomenological attitude, aiming to understand the lifeworld as presented by the participant^(16,21)^. The interviews were audio-recorded and later transcribed, with an average duration of 13 minutes. They were not returned to participants for review. Field notes were taken after each encounter. There were five refusals, justified by the presence of family members, lack of time, and distrust. No repeated interviews were conducted.

Data collection was concluded upon reaching theoretical saturation, a conceptual tool that allowed the suspension of new participant inclusion once the phenomenon was revealed and the lived experience typified based on the collected information and materials^(16,22)^. In this sense, data collection ended when no new elements were identified and the researcher’s inquiries had been addressed^(22)^.

### Data analysis

**Figure 1:**
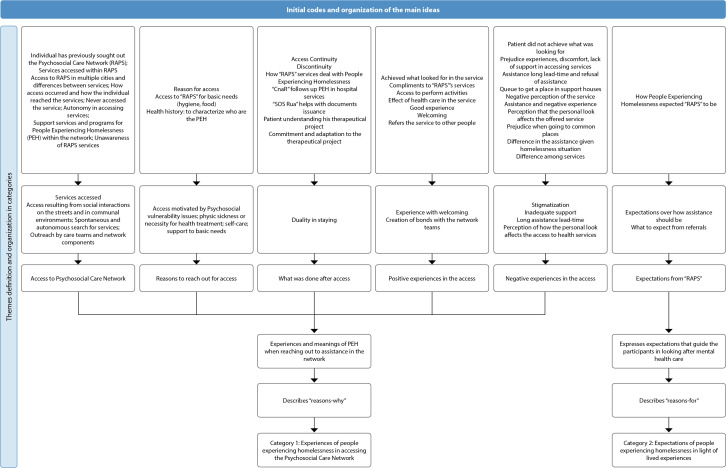
Illustration of the Stages of Coding, Naming, and Organizing Categories in the Analysis Process

The data collected were analyzed by the lead researcher without the use of software, followed by consensus with the other authors who contributed to the study to ensure its validity and reliability. Based on the theoretical and methodological framework, the lived type of the PEH’s experience in accessing the *“RAPS”* was constructed^(16)^. The recommended steps for data analysis were followed^(23)^: full transcription of the audio recordings, repeated, careful, and continuous readings of the narratives to grasp the meaning of the experiences reported by participants, coding of the narratives into units of meaning, and organization of these units into thematic categories (Figure 1) that illustrate the motivations experienced by the participants^(16,23)^. Considering that the description of the subject’s lived experience is the pathway to understanding the phenomenon, excerpts from the narratives were examined to enable the grouping of meaning units and the construction of thematic categories^(16)^. A meeting was held to present the study’s findings to the “*CnaR”* team, and the contributions were considered valuable for incorporation into daily care practices. No direct feedback was provided to the PEH participants.

## RESULTS

A total of 21 individuals experiencing homelessness participated in the study, including 18 men and 3 women, predominantly of mixed-race and Black backgrounds, ranging in age from 24 to 55 years. The duration of access to the *“RAPS”* varied, encompassing both initial experiences and long-term follow-up reports of up to ten years.

The lived type was organized into two thematic categories: “Experiences of people experiencing homelessness in accessing the Psychosocial Care Network”, which addresses the *reasons-why* behind the experiences and meanings attributed by the participants, and “Expectations of people experiencing homelessness in light of lived experiences”, reflecting the *reasons-for* that guide their pursuit of mental health care. The grouping of experiences within these categories enabled the unveiling of the phenomenon, and the findings were discussed in light of social phenomenology and scientific literature.

### Experiences of people experiencing homelessness in accessing the Psychosocial Care Network

In the context of psychological distress inherent to life on the streets, participants shared their experiences with *“RAPS”* services, as well as the means of access and the motivations that led them to seek these points of care. Their accounts highlighted a variety of services, including *“CnaR”*, “*Unidades Básicas de Saúde”* (*“UBS”*, Basic Health Units), *“Centros de Atenção Psicossocial”* (*“CAPS”*, Psychosocial Care Centers), *“Centro de Referência Especializado para População de Rua”* (*“Centro POP”*, Specialized Reference Centers for the Homeless Population), *“Serviço de Orientação Social a Pessoas em Situação de Rua”* (*“SOS Rua”*, Social Guidance Services for People Experiencing Homelessness), and shelter units described by participants as hostels, in addition to general hospitals and *“UPA”*.

*Yes, I’ve been able to access all the services I look for, whether it’s “CAPS”, “CnaR”, “Centro POP”, or basic health units.* (P3)

*Yes. “SOS” and the “CnaR” staff.* (P5)

*The network offers these shelters to help the individual, the user, move forward.* (P3)

*I’ve already been to the “UPA”, Ouro Verde Hospital, and the street doctors.* (P20)

Regarding strategies for accessing services, testimonies illustrated that contact with *“RAPS”* often arises from social interactions on the streets and in communal spaces such as lines at popular restaurants, public squares, and shelters. In these settings, the PEH share knowledge, stories, and experiences related to the care they’ve received within the network. The dissemination of information and recommendations for mental health services revealed a spontaneous pursuit of assistance and support.

*On the street, in the line at Bom Prato* […] *I was venting socially to someone in line. Then he guided me, even about aid and things I didn’t know about. Where did I go first? I went to “Centro POP”.* (P4)

*I was sitting there and suddenly the guy said: the “CnaR” is over there, that yellow van.* […] *So, I went over and started talking.* (P9)

*I was in front of the citizenship office, and they called me, ‘let’s go check out “CAPS”’. I said, ‘let’s go’.* (P11)

*I also give directions to people who don’t know the city, who don’t know any care or protection network* […] *I say, ‘look, go this way, it’s on this street, and talk to this person’.* (P3)

Moreover, many participants became aware of and accessed services through outreach efforts by care teams, mentioning *“SOS Rua”* and *“Centro POP”* as key facilitators in coordinating and referring them to other services within the network.

*I was experiencing homelessness* […] *and I was approached by the “SOS Rua” team* […] *they walk around the whole city, reaching out to people who are on the streets, sleeping outside. They help with documents too, and refer people to shelters, to “CAPS”* […] *They refer you to hostels.* (P5)

*Through the” Centro POP” service, which is connected with other teams, I was able to get that referral.* (P3)

*Through a social worker who’s currently following my case. She recommended that I come here.* (P18)

Access is usually driven by social vulnerability, substance dependence, harm reduction needs, and the necessity for other health treatments. Interviewees also mentioned seeking out the network for appointments with social workers, internet use, and a personal desire for self-care and transformation of their lived reality.

*A social worker appointment, there’s internet access too, but it’s really about self-care, to reduce or minimize the harm for someone experiencing homelessness, someone in social vulnerability, mainly due to substance dependence.* (P3)

*Because of the drugs and* […] *the desire for change too.* (P4)

*When I feel that urge to use, I ask them to refer me to “CAPS” so I can spend the day there.* (P5)

*All my vaccinations are done with them. Treatment for uric acid too.* (P12)

*Then I need to get an IV to feel better, stay in a bed for a while before surgery, so I can go a few days without drinking, until I recover.* (P6)

In the context of self-care vulnerabilities and immediate survival needs, participants reported seeking services to support hygiene, shelter, and food. Many prioritized this type of support over other forms of care offered by the network.

*Yes, because it’s a matter of self-care* […] *like, basic hygiene and food needs.* (P3)

*They do good work at “Centro POP”. They give you a little kit, and a voucher for a free lunch. It’s like a hygiene kit, it includes a meal, soap, toothpaste, a toothbrush, and toilet paper.* (P4)

*Getting treatment* […] *few people want treatment* […]. *Most just go there to eat and keep living the same way.* (P7)

Regarding continuity in mental health care services, interviewees emphasized ongoing access and follow-up within *“RAPS”*. They shared that they organize themselves to return to the units and ensure continuity through previously arranged agreements.

*Every Friday I’m here. Doing everything* […] *seeing the doctor.* (P21)

*So, with what little we can organize, we’ve been seeking access and we’ve been managing to get it, right?* (P3)

*Sometimes, just yesterday, I was at “CAPS”, and tomorrow I’ll be there again.* (P4)

*On Monday, right? I’ll go back other times, because they asked me to, the ones who are going to follow my case more closely.* (P8)

Other participants reported discontinuity in care, whether due to personal commitments, the perception that they did not need mental health assistance, or negative experiences that led to resistance in seeking help.

*These days I haven’t been going, because I’ve been dealing with some problems. Working over in Souzas, I just haven’t been able to go. It’s been, what* […] *about three or four months since I last went.* (P13)

*There was a time when I thought about leaving “CAPS”* […]. *Why did I want to switch “CAPS”? Because I felt like I wasn’t getting the attention I needed. You know when you feel, I don’t know* […] *neglected.* (P4)

Among the negative experiences described, the PEH reported facing inadequate support, discrimination, and prejudice, which led to feelings of exclusion, neglect, and discomfort. They also noted that discomfort is heightened when using services accessed by individuals from other social realities, such as *“UBS”* and hospitals. According to the interviewees, this scenario does not apply to *“CnaR”*, as they recognized in this mobile service a team that provides differentiated and respectful treatment to the PEH.

*If you have an accident and you’re with me, even living the life you live, you’ll be treated the same as me. You don’t have ID, no phone. ‘When we get to it, we’ll see’. That’s how it works. ‘I’m closing’.* […] *That’s just how it is.* (P1)

*There’s something, I don’t know how to say it, I can’t call it prejudice.* […] *But there’s a certain discomfort when accessing these services, because there are other people from different social realities using the same service at the same time. And we feel a bit uncomfortable, but maybe that’s just our perception too, right?* (P3)

*People* […]. *They just look down on us* […]. *That’s all.* (P16)

*To be treated differently? Here* [“CnaR”]*. Because in the other health systems, no. Here, I don’t know if they’re trained or what. But it’s a different kind of care, eye to eye, asking what you need. They really listen, talk to you, everything.* (P1)

According to the interviewees, this stigmatization was reflected in judgmental attitudes, longer waiting times for assistance, and unsatisfactory care. Negative experiences were further intensified when the PEH wore soiled clothing or carried personal belongings.

*I sat on that bench for three hours, waiting to be seen* […] *crying and rolling on the ground. Because of being homeless. A lot of people came and went ahead of me. I kept getting pushed back. And they judge, right? A lot of people judge.* (P11)

*Because they say, ‘he’s homeless,’ nobody cares* […] *‘You live on the street, you can wait for hours and hours. We’re not going to see you now.’* […] *Other people who are well dressed, who have money, they get seen quickly* […] *Homeless people, they can sit there for a week and still get poor treatment. They don’t treat us properly.* (P13)

*I was in a different situation, I was poorly dressed, carrying blankets and stuff. So, whether we like it or not, even culturally, that makes people perceive us differently. Our reality.* (P3)

Although PEH face adverse and undesirable situations, some interviewees emphasized positive experiences within the services, such as receiving quality care. They also referred to the possibility of reducing drug use through dialogue and relationship-building with care teams in the network.

*That part of the care at “CnaR”, “CAPS”, and the hospitals has been good. They treat us well, everything.* (P13)

*For me, all the ones I’ve been to are really great, you know?* […] *The street doctors, “SOS Rua”. For me, they’re excellent.* (P20)

*The care is wonderful. Kindness. You can tell they treat us like people.* (P21)

*Oh yeah, they treat us really well. They’re very respectful* […] *Especially the doctor* […] *When someone’s out of it, lying there, high, under a tree - he’ll go over, kneel down next to the person, talk* […]. *They’re amazing people.* (P15)

*She comes here just to talk to someone. To hear someone’s voice, to not hear that she’s ‘trash’, dirty, a ‘junkie’* […]. *To not use drugs or just to drink a glass of water, talk to someone. Breathe* […] *Because this place makes us feel human.* (P1)

Regarding the available services, testimonies illustrated the importance of *“CnaR”* in providing health care to PEH, as the care is fast, fluid, respectful, and does not require appointments. Finally, because it is a space specifically designed to focus attention on PEH, feelings of being welcomed were frequently mentioned.

*I think a lot would be missing, you know? If this work didn’t exist, I think people living on the streets would really suffer.* (P10)

*If it weren’t for “CnaR” today, I probably wouldn’t be able to get a prescription, I’d have to schedule an appointment at a “UBS”, which would take two months. So sometimes, people experiencing homelessness actually have certain advantages that the general population doesn’t. A regular appointment takes two months, but I come here and I’m seen in ten minutes.* (P3)

*And the best thing about “CnaR” is that it treats homeless people just like anyone else* […]. *There’s none of that ‘oh, you smell because you live on the street’* […]. *You know? It’s wonderful.* (P21)

*“CnaR” is a service, I mean* […] *it has a purpose, and that purpose is fulfilled, you know? They’re very kind, very attentive. This service really makes all the difference.* (P3)

### Expectations of people experiencing homelessness in light of lived experiences

The experience of PEH in accessing services within the network is complex and multifaceted, generating expectations that reflect their aspirations and the challenges they face. In light of this, the group expressed a desire for equal and universal care that guarantees everyone the right to health services. Therefore, the PEH reported wanting health professionals to treat all individuals uniformly, regardless of their social or personal characteristics.

*But it shouldn’t be different. Because the Sistema Único de Saúde* [“SUS”] *is the Sistema Único de Saúde. It’s for everyone.* (P1)

*I expected it to be about equality; we’re citizens just the same. We have a “CPF”* [taxpayer ID] *like everyone else, we bleed the same color, you know? Our blood is red* […]. *That day, I expected to be treated like a ‘normal’ person, in quotes, because we’re all normal, each with our own characteristics, right?* (P3)

*We shouldn’t be choosing who we treat, right?* (P1)

*Because they treat one person one way, another person another way. One gets treated badly, another gets treated well* […]. (P13)

Participants also expressed a desire to be recognized as people, not as statistics or mere numbers in service records. They long for care that is welcoming, empathetic, and humanized so that the specific needs of their group are truly acknowledged and addressed.

*I don’t want to be a statistic. I want to be welcomed; I want to be heard.* (P1)

*If someone isn’t humane, they won’t pay attention to others, right? Because nowadays people are mostly thinking about themselves, not about others.* (P11)

The PEH expressed the need for prompt care and the desire to access shelter units and beds, recognizing their role in the transition from street life back into society. According to the interviewees, access to shelters enabled them to organize their thoughts, financial matters, and social issues-factors considered essential for social reintegration and the restoration of dignity.

*There needs to be something a bit more agile to help resolve this situation , and sometimes, to take a closer look at the mental state of the person going through it.* (P10)

*I’m not on the streets because I want to be. I’m on the streets because I’m going through hardship. If I could get even just a room, that would be something. Right? That would change everything.* (P14)

*It’s another service too* [Shelter Unit]*, I’m not sure if it’s part of the “RAPS” network, but it’s a service that’s really going to help me in this transition from street life to readjustment, to returning to society. So, I’ll need this period to stabilize, to get that support from the shelter, so I can get organized, you know? Organize my thoughts, feelings, financial and social matters, until I reach financial stability, especially social stability, so I can have autonomy.* (P3)

*I think it’s about restoring dignity, right?* (P3)

In contrast, the PEH reported having received care in the way they had hoped by being treated with respect and hospitality, in a manner that met their expectations.

*In my mind, I expected it to be like this. Well treated, treated like a human being. With respect, with kindness. Welcomed. You feel embraced by them.* (P7)

*I don’t think anything needs to change. It’s perfect. I think there should be more of it. Because here it’s better than* […] *you know, in terms of care. The care is wonderful, I think. I have nothing to complain about. Not about the team, nothing, you know?* (P21)

## DISCUSSION

The approach to the lifeworld of the PEH enabled an understanding of their access to the “*RAPS”* services, allowing for the apprehension of the intentions and expectations that guide this population in seeking care within the network, based on their lived experiences^(16)^. Despite the presence of various barriers, it was observed that the PEH participants in the study move between the available points of care within the municipality’s *“RAPS”*. In this regard, they mentioned access to psychosocial care services, primary care, as well as emergency and urgent care services, hospital services, and temporary residential care. This finding contrasts with the literature, which indicates that PEH generally only use emergency and urgent care services during acute situations^(13,14,24)^.

The access experiences reported by the PEH participants occurred due to the structure of the *“RAPS”* in the municipality where the research was conducted, as it includes care and reception by the *“CnaR”* teams, which understand the particularities of the PEH and the dynamics of the territory in which they circulate. It is noteworthy that the *“CnaR”* enhances this population’s accessibility to health services by coordinating demands and promoting comprehensive care, while its absence reduces the mobility of PEH, especially in areas without primary care coverage or *“CnaR”* teams^(13,19)^. As a contribution of this study, it is highlighted that the absence of health services compromises the mobility, care, and access of PEH to the network.

Among the strategies used by PEH to access *“RAPS”* services, testimonies described contact with the network arising from interactions in public spaces such as streets, popular restaurants, squares, shelters, and other locations. In these environments, PEH share their knowledge and recommend mental health care services to one another based on places where they previously had positive experiences. This finding highlights the importance of outreach strategies adapted to PEH, carried out in public spaces where professionals, through informal interactions^(25)^, build relationships of trust and empathy, which foster bonds and reduce barriers to accessing mental health care^(26)^. These strategies are guided by the body of knowledge and lived experiences of PEH, accumulated throughout their life trajectories, and by face-to-face relationships characterized as authentic encounters in which individuals share time and space, becoming mutually aware^(16)^.

The sharing of experiences and recommendations for care locations enables PEH to seek assistance services more autonomously, while teams such as “*SOS Rua*”, *“CnaR”*, and “*Centro POP*”, through integrated actions, facilitate their mobility and ensure referrals within the network. This evidence aligns with the literature that emphasizes the importance of PEH’s accessibility to health services and social assistance institutions, supported by matrix support actions, specialized teams^(13)^, more flexible service hours, and attentive care to prevent PEH from being neglected^(27)^. The work of these teams, using strategic approaches within the everyday lifeworld of PEH and through face-to-face care relationships, can minimize access barriers, provide welcoming support, and assist this population in recognizing their needs^(13,16)^.

According to the interviewees, the search for care within the network is driven by the implications of life on the streets. The literature indicates that PEH primarily seek emergency and urgent care services in response to demands arising from their reality, in which health is associated with the absence of disease and functional capacity^(14,28)^. However, an increase in the demand for primary care was also identified, which may be a result of the high prevalence of chronic illnesses and the aging of PEH population, making it necessary to ensure access to health promotion and prevention services^(29)^. Additionally, the PEH reported accessing the network for appointments with social workers, motivated by a desire to transform their lifeworld, as well as to use the internet. The use of technology to promote health among this population reinforces the idea that connectivity enables communication with friends and family, highlighting its influence on mental health^(10,30,31)^.

Interviewed PEH pointed out that, in many instances, they prioritize care for immediate needs - such as hygiene and food security - over other services offered by the *“RAPS”*. This scenario stems from the multiple dimensions of vulnerability experienced by this population^(8)^. Literature provides evidence supporting the need for intersectoral collaboration^(32)^ and cooperative approaches^(33)^ in the field of mental health, aiming to improve actions for comprehensive care and to better understand the unique characteristics of PEH^(32,34)^.

The findings illustrate the contrast between continuity and discontinuity in service use, with interruptions being associated with personal commitments, the perception of not needing mental health care, and negative experiences within *“RAPS”*. Treatment abandonment occurs in both high-income (30%) and low-income countries (45%)^(35)^ and is linked to the complexities of the healthcare system, negative access experiences, and lack of trust in professionals^(25)^.

Among the challenges faced when accessing the *“RAPS”*’s services, the PEH reported inadequate support, prejudice, and discrimination, which lead to feelings of exclusion and discomfort. They also mentioned that discomfort is heightened in services frequented by individuals from different social realities. This suggests that health services are not adequately prepared to receive and accommodate the unique needs of PEH, making care for vulnerable groups a persistent challenge^(13,28,36)^. Furthermore, accessibility to mental health services may be compromised by professional practices influenced by stereotypes and negative judgments^(13)^.

Negative experiences are intensified when PEH access the network carrying personal belongings and with impaired hygiene, due to limited access to basic sanitary conditions^(13,14,28)^. From a phenomenological perspective, these belongings may reflect unique meanings tied to their life history - for example, a blanket may symbolize both physical and emotional shelter^(16)^. Such negative experiences hinder care and become barriers to access, contradicting the principles of universality and equity of Brazil’s “*SUS”*, as well as the goals of the *“PNPSR”* ^(7,13,14)^.

As an undesirable experience, the PEH also referred to the prolonged waiting time for assistance, which, from their perspective, is linked to stigmatization. It was noted that extended stays in care units cause anxiety, as individuals worry about activities related to securing their subsistence^(15)^. This highlights that humanized care for PEH is still overshadowed by persistent barriers, marked by prejudice and stigma present in other *“RAPS”* services^(13)^.

According to the participants, access to the network can help reduce the use of psychoactive substances, as it provides opportunities for welcoming and dialogue with professionals during moments of vulnerability. It is emphasized that the use of soft technologies is essential in mental health care, as they foster bonds and authentic encounters that connect subjectivities through face-to-face relationships^(13,16)^, such as the “peer support” strategy, used in harm reduction and led by individuals with experiences similar to those of PEH^(37)^.

Positive experiences were also highlighted, especially in relation to the *“CnaR”*. Participants emphasized the importance of having *“CnaR”* services available in the municipality for mental health care, noting that these services offer fast, fluid assistance without the need for scheduled appointments or exams. They also stated that, in these settings, they do not feel excluded by the staff. This type of care is particularly relevant for PEH, as the requirement for scheduled times and waiting in lines can interfere with their access to daily meals, due to overlapping hours with popular restaurant services, for example^(38)^. Considering that food insecurity can compromise continuity of care^(38)^, the importance of services that combine welcoming approaches, flexibility, and accessibility in mental health care, such as those provided by *“CnaR”*, is reinforced.

The PEH also expressed various intentions, such as receiving equal and universal care in which health professionals treat everyone with consistency and humanization. They seek to be recognized as people, not as statistics or service counts. Therefore, it is essential that professionals adopt welcoming and empathetic practices in caring for PEH, contributing to the development of therapeutic pathways that are sensitive to the specificities of the health-disease process^(39)^, by recognizing the individual as a social actor embedded in their lifeworld^(16)^.

*“RAPS”*’s services still face challenges in providing care with equity and empathy, due to the persistence of stigmatized and stereotyped conceptions. This underscores the need to train professionals to act with sensitivity toward the complexities involved in mental health care, as well as the urgency of promoting ongoing team qualification processes through continuing education aimed at building inclusive and transformative practices^(5)^. This desired professional profile is reflected in the interviewees’ accounts, as they expressed a longing for care marked by empathy, humanization, and recognition of their individual circumstances.

Based on their lived experiences within the network, another expectation expressed by the PEH is access to shelter units and beds, emphasizing the importance of having a refuge for their health and reintegration into society. Having shelter supports the process of psychosocial and financial organization and helps restore dignity. This relationship can be observed through the social determinants of health model, which understands health as a socially constructed phenomenon, shaped by historical and structural processes such as labor, class relations, and public policies^(40)^, all of which permeate the individual’s lifeworld and directly influence their health^(16)^. In this sense, the lack of temporary shelter spaces and inadequate housing conditions negatively affect the health-disease process of PEH, while their existence, on the other hand, promotes social reintegration and increases the likelihood of formal employment, an essential factor for successful reintegration^(11)^.

Finally, many interviewees reported that the care they received met their expectations, characterized by hospitality and respect. Health care, regardless of who is being assisted, must take into account the social context and be delivered by professionals who recognize the individual as a subject of rights, an essential aspect of the professional-patient relationship^(19)^. This broader perspective may arise from intersubjective relationships, in which PEH are acknowledged and their experiences, biographical circumstances, and body of knowledge are valued^(16)^.

### Study limitations

Regarding limitations, it is emphasized that the research was restricted to a single municipality - an area with active “*CnaR”* services - where territorial boundaries, local characteristics, and service availability may influence the dynamics of access and care observed in other contexts.

### Contributions to the field of study

As a contribution to the field of nursing, this study expands the understanding of PEH’s access to *“RAPS”*’s services and provides support for nurses to act with sensitivity, critical awareness, and commitment. By understanding the motivations and meanings attributed to PEH’s actions, nurses can enhance their listening skills, adapt care strategies, and strengthen therapeutic bonds. Therefore, by unveiling the phenomenon through the lens of those who experience it, the study aims to contribute to the social visibility of PEH and amplify their voice in society. Furthermore, the importance of the *“CnaR”* is emphasized, as this service enables new dynamics of access to the network for those who live the street as part of their lifeworld.

## FINAL CONSIDERATIONS

This study enabled an understanding of access to *“RAPS”* from the perspective of the PEH in a municipality in the interior of São Paulo. It was observed that this access is shaped by lived experiences on the streets, molded by vulnerability and the biographical situation of PEH, elements that constitute the “reasons-why”. The “reasons-for” reveal the expectations and life projects of the interviewees, pointing toward desired future directions within their biographical trajectory.

By bringing together and understanding the existential motivations of this population, a typical pattern of action emerges: PEH demonstrate significant mobility within the network, facilitated by the work of *“CnaR”* and the coordination of care teams. Access occurs through social interactions in shared spaces, where knowledge about services is exchanged. However, the duality between continuity and discontinuity in care was identified, linked to lived experiences. The PEH highlighted welcoming and quality care but also reported discrimination and inadequate support in services that cater to diverse social realities.

Among their expectations, the desire for equal and universal care was emphasized, along with the need to be recognized as individuals rather than statistics. To meet these demands, the importance of welcoming, empathetic, and sensitive practices by health professionals is reinforced - practices aligned with the principles of Brazil’s *“SUS”* and *“PNPSR”*. In light of the barriers and stigma faced, further studies are recommended, focusing on professional practices and on strengthening and qualifying access to the network.

## Data Availability

The research data are available within the article.
