# Supplementary material for: Access to mental health services: the perspective of people experiencing homelessness
Source: Rev Bras Enferm. 2026 Jul 24;79:e20250045. doi: 10.1590/0034-7167-2025-0045 (PMC13403642; doi:10.1590/0034-7167-2025-0045)
Supplement: Translation

# Translation: Acesso a serviços de saúde mental: perspectiva da população em situação de rua

**DOI:** 10.1590/0034-7167-2025-0045pt

**Authors:** Tatiane Cristina da Silva, Aldair Weber, Giulia Delfini, Vanessa Pellegrino Toledo

**Affiliations:** I Unversidade Estadual de Campinas. Campinas, São Paulo, Brasil

**Keywords:** População em Situação de Rua, Saúde Mental, Serviços de Saúde Mental, Acesso Efetivo aos Serviços de Saúde, Atenção à, Saúde.

## Abstract

**Objetivos:**

compreender o acesso da população em situação de rua aos serviços de saúde mental da Rede de Atenção Psicossocial em um município do interior paulista.

**Métodos:**

estudo qualitativo, baseado na sociologia fenomenológica de Alfred Schutz, com 21 pessoas em situação de rua entrevistadas em julho de 2024. A análise desvelou duas categorias: “Experiência da população em situação de rua no acesso à Rede de Atenção Psicossocial” e “Expectativas diante das experiências vividas”.

**Resultados:**

a mobilidade entre os pontos de atenção da rede é motivada por barreiras no cuidado em saúde mental e a presença do Consultório na Rua facilita o acesso. Observa-se tanto permanência quanto descontinuidade no cuidado associadas à experiências de acolhimento e também de discriminação. As expectativas incluem assistência igualitária e universal.

**Considerações Finais:**

o acesso, marcado por vulnerabilidade, suporte inadequado, discriminação e tensão pela ruptura do cuidado, requer acolhimento, qualificação da assistência e da atuação profissional.

## INTRODUÇÃO

Globalmente, estima-se que 150 milhões de pessoas vivam em situação de rua^(1)^. No Brasil, segundo dados do Cadastro Único para Programas Sociais (CadÚnico), foram registradas aproximadamente 221 mil entre 2018 e julho de 2023^(2,3)^. Campinas, no interior paulista, está entre os dez municípios que concentram 48% da População em Situação de Rua (PSR) no país, registrando 1.905 pessoas em 2021 e 2.547 em 2023, ainda de acordo com o CadÚnico^(2)^. Contudo, dados da Secretaria Municipal de Assistência e Desenvolvimento Social do município indicaram 932 pessoas nas ruas em 2021 e 1.300 em 2024, revelando discrepâncias na representação da realidade dessa população^(4,5)^.

Internacionalmente, a situação de rua é considerada um fator resultante de desigualdades estruturais, pobreza, exclusão social e ausência de políticas integradas^(6)^. No Brasil, a Política Nacional para a População em Situação de Rua (PNPSR)^(7)^, de 2009, caracteriza a PSR como um grupo heterogêneo e vulnerável socialmente, que compartilha em suas vivências a pobreza extrema, vínculos familiares e afetivos fragilizados e a inexistência de habitação convencional regular^(7)^. Nesse sentido, compreende-se a vulnerabilidade como um fenômeno único e complexo resultante da interação de aspectos das dimensões individuais, sociais e programáticas, sendo esse conceito empregado para compreender as suscetibilidades que expõem as pessoas a problemas e agravos de saúde^(8)^. A situação de rua, por sua vez, é resultado do modo de produção vigente, da exclusão do mercado de trabalho, da urbanização desordenada, das crises econômicas cíclicas e da profunda desigualdade social^(9)^.

Nesta realidade, a PSR utiliza logradouros públicos, unidades de acolhimento e áreas degradadas e abandonadas como locais de abrigo permanente ou provisório, e busca nesses espaços meios de subsistência^(10)^. O fenômeno da vivência em situação de rua emerge de profundas desigualdades sociais, manifestando-se por meio de fatores como desemprego, precarização das condições de vida, uso abusivo de álcool e outras drogas, rupturas de vínculos familiares, migrações, retorno do encarceramento, ameaças à segurança nos locais de origem, decisões pessoais e problemas de saúde física e mental^(9,10)^.

A PSR enfrenta a vulnerabilidade em seu cotidiano por meio da ausência de abrigos seguros e por dificuldades de acesso à alimentação, higiene, descanso e cuidados de saúde essenciais, expondo-se a incertezas constantes e à precariedade extrema^(9,10)^. Esse contexto intensifica sua exposição a fatores de risco e favorece a incidência e o agravamento de transtornos mentais^(11)^. Tais condições estão associadas a fatores socioeconômicos, à fragilidade das redes de apoio, à baixa escolaridade, ao desemprego e a vínculos sociais enfraquecidos, resultando em altas taxas de depressão, bipolaridade, esquizofrenia e dependência química^(11,12)^. A sobreposição entre a vivência nas ruas e o sofrimento psíquico aumenta a exclusão social, dificulta o acesso aos serviços de saúde e compromete a continuidade do cuidado, acentuando a vulnerabilidade dessa população^(9-12)^.

Diante disso, as políticas públicas no Brasil, tal como a PNPSR^(7)^, buscam garantir atenção integral, fortalecer ações do Sistema Único de Saúde (SUS) e assegurar o acesso amplo, comunitário e seguro aos serviços das redes de saúde, como a Rede de Atenção Psicossocial (RAPS)^(7)^. Contudo, os princípios da universalidade e equidade do SUS permanecem distantes da PSR, cujo acesso à saúde é irregular e, em geral, motivado por emergências que comprometem sua sobrevivência^(13)^. Nesses casos, a busca por atendimento ocorre, em sua maioria, nas Unidades de Pronto Atendimento (UPA), em detrimento de serviços da atenção básica que desenvolvem ações preventivas e de promoção à saúde^(13,14)^.

Nesse cenário, o acesso da PSR a serviços de atenção psicossocial especializada, como o Consultório na Rua (CnaR), é limitado, a depender do apoio matricial e da intermediação de instituições socioassistenciais^(15)^. Além disso, a mobilidade territorial de parte da PSR pode representar um desafio para a organização de serviços de saúde com base em delimitação geográfica e população adscrita, afetando o planejamento de ações e o acompanhamento contínuo^(5,14)^. Essa dinâmica resulta em cuidados fragmentados e descontínuos, o que impacta negativamente a saúde mental ao fragilizar vínculos terapêuticos, redes de confiança e o acolhimento^(5,14)^.

Nesse contexto, a vulnerabilidade e o crescimento contínuo da PSR exigem a produção e a sistematização de dados oficiais consistentes sobre a realidade vivida por este grupo populacional que subsidiem a formulação de políticas públicas efetivas e coerentes com os princípios da PNPSR, pois a ausência de informações precisas compromete o dimensionamento real dessa população e dificulta o planejamento de ações intersetoriais^(3-5)^.

Dessa forma, este estudo justifica-se pelo cenário demográfico e em razão do contexto vivido pela PSR nos quais o acesso esporádico e descontínuo aos serviços de saúde dificulta o planejamento de ações eficazes para esta população^(7,13-15)^. Essas barreiras afetam a saúde mental da PSR, comprometendo a construção de vínculos terapêuticos e de redes de confiança, essenciais para o cuidado integral^(13-15)^. Tais aspectos reforçam a necessidade de estudos que aprofundem a compreensão sobre as dinâmicas de acesso e cuidado da PSR aos serviços de saúde, o que possibilita espaço para a vocalização do fenômeno na percepção de quem o vivencia.

## OBJETIVOS

Compreender o acesso da PSR aos serviços de saúde mental da RAPS de um município do interior paulista.

## MÉTODOS

### Aspectos éticos

O estudo foi aprovado pelo Comitê de Ética em Pesquisa da Universidade Estadual de Campinas no ano de 2024 e seguiu as recomendações éticas de acordo com a Resolução n. 466/12 do Conselho Nacional de Saúde. Os participantes assinaram o Termo de Consentimento Livre e Esclarecido, consentiram por gravação em áudio e foram identificados pela letra P, seguida de numerais arábicos, de forma a garantir sigilo e ordem cronológica às entrevistas.

### Referencial teórico-metodológico

Alfred Schütz propõe a compreensão dos motivos das vivências anteriores (“motivos-porque”) e das intenções orientadas para ações futuras (“motivos-para”), os quais impulsionam e projetam os sujeitos em seu mundo-vida^(16)^. Esse é entendido como um cenário intersubjetivo habitado pelo homem, que desenvolve suas ações, estabelece interações e atribui significados por meio das relações face a face e de seus aspectos biográficos que, juntos, constituem seu acervo de conhecimentos^(16)^. Dentre as diferentes tipificações existentes na sociologia fenomenológica, o tipo vivido refere-se às ações no mundo social, interpretadas e organizadas com base nas motivações^(16)^. A partir dessas experiências, constroem-se padrões comuns de significado que auxiliam as pessoas a compreenderem e interpretarem a realidade compartilhada^(16)^.

### Tipo de estudo

Estudo de abordagem qualitativa, norteado pelo *Consolidated Criteria for Reporting Qualitative Research - COREQ* ^(17)^ e apoiado no referencial teórico-metodológico de Alfred Schütz^(16)^. Tal abordagem possibilita uma compreensão profunda do acesso da PSR aos serviços de saúde mental, pois permite desvelar a essência do fenômeno ao considerar a rua como seu mundo-vida, permeado por vivências, motivações, aspectos biográficos, acervo de conhecimento, experiências intersubjetivas da vida cotidiana e significados atribuídos às suas ações^(16)^.

### Cenário de estudo

A pesquisa foi conduzida em um município do interior paulista com cerca de 1,1 milhão de habitantes, com infraestrutura urbana desenvolvida e instituições de referência, além de polos industriais, tecnológicos e de serviços^(18)^. Nesse cenário, a cidade registra elevado índice de PSR, apesar das contradições sobre os dados de indivíduos que habitam as ruas da cidade^(2,4)^.

Participaram deste estudo pessoas em situação de rua assistidas pelo CnaR do município, ponto de atenção da RAPS com atendimento itinerante direcionado ao suporte dessa população^(19)^. Conduzido por equipe multidisciplinar, o CnaR desenvolve ações de promoção e prevenção à saúde voltadas às demandas da PSR, com atuação integrada em unidades básicas, serviços especializados e equipes de vigilância em saúde do território^(20)^. As três equipes do CnaR realizam aproximadamente 800 atendimentos mensais^(20)^. Contudo, este estudo abordou somente uma equipe, responsável pelas ações na região central da cidade, estrategicamente situada junto à maior concentração da PSR. A equipe realiza atendimentos em unidades móveis em dois pontos fixos na região com o objetivo de consolidá-los como espaços de referência para a PSR acessar o serviço.

### Fonte de dados

Os critérios de inclusão foram: ter no mínimo dezoito anos, estar em situação de rua e em acompanhamento no CnaR, enquanto serviço de saúde mental da RAPS, no momento da coleta de dados. O tempo de permanência nas ruas não foi utilizado como critério, visto que o fenômeno foi compreendido nos aspectos subjetivos da experiência da PSR, independentemente da temporalidade em que foi vivenciada^(16)^. O critério de exclusão da pesquisa foi o de indivíduos que apresentaram dificuldade de verbalização devido a efeitos medicamentosos ou por desorganização psíquica.

### Procedimentos metodológicos, coleta e organização dos dados

Os pesquisadores reuniram-se com a coordenação do CnaR para alinhar o início da coleta de dados, realizada em junho e julho de 2024 pela primeira autora, graduanda de enfermagem à época, com acompanhamento da equipe de pesquisa para garantir sua adequada condução. A referida pesquisadora, além de experiência de atuação com a PSR, também realizou entrevistas piloto, que foram transcritas e analisadas no intuito de qualificar a técnica de coleta aplicada. Nesse período, a pesquisadora apresentou-se à equipe do CnaR e frequentou o serviço para conhecer a dinâmica de trabalho e estabelecer contato inicial com a PSR. Após esse momento, iniciou a coleta de dados nos pontos fixos de ações do CnaR e convidou para participarem da pesquisa as pessoas que atendiam aos critérios de inclusão, explicando a elas qual seu objetivo. A equipe do CnaR permaneceu no local durante as entrevistas, porém não houve interferências. Utilizou-se a amostragem por conveniência e as entrevistas foram realizadas em dois pontos fixos, praças públicas onde o serviço dispunha de mesas e cadeiras ao ar livre, próximos à unidade móvel, com o objetivo de acolher a PSR.

Foram realizadas entrevistas fenomenológicas, individuais, não diretivas e semiestruturadas^(21)^, com a questão norteadora “Como você acessou os serviços de saúde mental?”, e dessa surgiram outras questões que possibilitaram o aprofundamento e a compreensão do fenômeno estudado: “Conte-me como foi a experiência de frequentar serviços de saúde mental?” e “Como você esperava que fosse esse atendimento?”. Durante a entrevista, os pressupostos da pesquisadora foram colocados em suspensão, com a adoção da atitude fenomenológica para compreender o mundo-vida como apresentado pelo participante^(16,21)^. As entrevistas foram gravadas em áudio e posteriormente transcritas, com duração média de 13 minutos. Essas não foram devolvidas aos participantes para revisão. As notas de campo foram realizadas após cada encontro. Houve cinco recusas, justificadas pela presença de familiares no ambiente, indisponibilidade de tempo e desconfiança. Não foram realizadas entrevistas repetidas.

A coleta de dados foi encerrada quando se alcançou a saturação teórica, ferramenta conceitual que possibilitou a suspensão da inclusão de novos componentes à pesquisa quando ocorreu o desvelamento do fenômeno e a tipificação do vivido a partir das informações e dos materiais coletados^(16,22)^. Desse modo, a coleta foi encerrada no momento que nenhum novo elemento foi identificado e as inquietações da pesquisadora foram respondidas^(22)^.

### Análise dos dados

Os dados coletados foram analisados pela pesquisadora principal, sem uso de *software* e com posterior consenso com os demais autores que contribuíram no estudo para garantir a validade e confiabilidade da pesquisa. A partir do referencial teórico-metodológico, construiu-se o tipo vivido da experiência da PSR no acesso a RAPS^(16)^ e seguiram-se os passos recomendados para a análise dos dados^(23)^: transcrição integral dos áudios, leituras repetidas, cuidadosas e contínuas dos discursos a fim de apreender o significado das experiências relatadas pelos participantes, codificação dos discursos em unidades de sentido, e organização dessas em categorias temáticas (Figura 1) que ilustram as motivações vividas pelos participantes^(16,23)^. Tendo em vista que a descrição da vivência do sujeito é o caminho para a compreensão do fenômeno, foram investigados nos discursos trechos que possibilitaram o agrupamento das unidades de sentido e a construção das categorias temáticas^(16)^. Foi realizado um encontro para apresentação dos resultados da pesquisa à equipe do CnaR e as contribuições do estudo foram consideradas importantes de serem inseridas nas práticas cotidianas de cuidado. Não ocorreu a devolutiva diretamente com a PSR.

**Figura 1:** Ilustração das etapas de codificação, nomeação e organização das categorias no processo de análise

## RESULTADOS

Participaram 21 pessoas que vivenciam a situação de rua, sendo 18 do sexo masculino e três do feminino, majoritariamente de cor parda e preta, com faixa etária entre 24 e 55 anos. O tempo de acesso à RAPS foi heterogêneo, abrangendo desde experiências iniciais até relatos de acompanhamento por até dez anos.

O tipo vivido foi organizado em duas categorias temáticas: “Experiência da população em situação de rua diante do acesso à Rede de Atenção Psicossocial”, que aborda os “motivos-porque” das vivências e significações da PSR, e “Expectativas da população em situação de rua diante das experiências vividas”, refletindo os “motivos-para” da busca por cuidados em saúde mental. A reunião de vivências nessas categorias possibilitou o desvelamento do fenômeno e os achados foram discutidos à luz dos conceitos da fenomenologia social e da literatura científica.

### Experiência da população em situação de rua diante do acesso a Rede de Atenção Psicossocial

No contexto de sofrimento psíquico inerente à vida nas ruas, os participantes compartilharam experiências com os serviços da RAPS, assim como os meios de acesso e as motivações que os conduziram a procurar esses pontos de atenção. Os relatos ressaltaram diversos serviços, como CnaR, Unidades Básicas de Saúde (UBS), Centros de Atenção Psicossocial (CAPS), Centro de Referência Especializado para População de Rua (Centro POP), Serviço de Orientação Social a Pessoas em Situação de Rua (SOS Rua) e unidades de acolhimento descritas pelos participantes como abrigos, além de hospitais gerais e UPA.

*Mas eu tenho conseguido, sim, acessar todos os equipamentos que eu procuro, qual foi o CAPS, qual foi o CnaR, Centro POP, unidades básicas de saúde.* (P3)

*Sim. O SOS e o pessoal do CnaR.* (P5)

*A rede dispõe desses abrigos para ajudar o indivíduo, o usuário, a seguir caminho.* (P3)

*Já frequentei a UPA, o Hospital Ouro Verde, e os médicos de rua.* (P20)

No que se refere às estratégias de acesso da PSR aos serviços, depoimentos ilustraram que o contato com a RAPS pode decorrer de interações sociais nas ruas e em ambientes de convivência, como filas de restaurantes populares, praças e abrigos, em que a PSR compartilha conhecimentos, histórias e vivências acerca de atendimentos vivenciados na rede. A difusão de saberes e indicações de locais para o cuidado à saúde mental demonstrou a busca espontânea por ações de assistência.

*Na rua, na fila do Bom Prato* [...] *eu me queixando socialmente na fila com alguém. Aí ele que me orientou, até negócio de auxílio, essas coisas que eu não tinha conhecimento. Eu fui aonde primeiro? Eu fui no Centro POP.* (P4)

*Eu tava sentado lá e daqui a pouco o cara falou assim: o CnaR tá ali, aquela van amarela.* [...] *Aí fui lá e comecei a conversar.* (P9)

*Tava na frente da cidadania, chamaram, vamo lá conhecer o CAPS. Eu falei, vamo.* (P11)

*Então eu indico para pessoas também que não conhecem a cidade, que não conhecem nenhuma rede de atenção* [...] *de proteção* [...] *eu falo, olha, vai por esse caminho, é aqui nessa rua tal e conversa com tal pessoa.* (P3)

Ademais, muitos participantes conheceram e acessaram os serviços por meio de abordagens de equipes assistenciais e mencionaram o SOS Rua e o Centro POP como auxiliares na articulação e nos encaminhamentos para os demais serviços da rede.

*Eu estava em situação de rua* [...] *E fui abordado por pessoal do SOS Rua* [...] *eles andam pela cidade toda, abordando as pessoas que estão em situação de rua, que estão dormindo na rua, aí fazendo documento também, encaminha para o abrigo, para o CAPS* [...]. *Encaminha para o albergue.* (P5)

*Através do serviço do Centro POP, interligado com outras equipes, eu consegui esse encaminhamento.* (P3)

*Através de uma assistente social que está acompanhando o meu caso agora. Ela me indicou a estar vindo aqui.* (P18)

O acesso usualmente é motivado pela vulnerabilidade social, pela dependência química, para redução de danos e por necessidade de outros tratamentos de saúde. Os entrevistados citaram ainda a busca pela rede para atendimentos com assistentes sociais, para o uso de *internet*, além do desejo individual de autocuidado e de transformação da realidade vivida.

*Um atendimento com assistente social, tem internet, mas é pelo autocuidado para ou reduzir ou minimizar os danos da pessoa em situação de rua, da pessoa em questão de vulnerabilidade social, devido principalmente à dependência química.* (P3)

*Por causa da droga e por* [...]. *O desejo de mudança também.* (P4)

*Dá aquela vontade de fazer o uso, hoje vou pedir para eles me encaminhar para o CAPS, para eu passar o dia lá.* (P5)

*Toda a minha vacinação é com eles. Tratamento do ácido úrico.* (P12)

*Aí eu tenho que tomar um soro pra me melhorar também, ficar um pouco de leito pra depois operar também, pra eu ficar uns dias sem beber também, até sarar.* (P6)

No contexto de fragilidades no autocuidado e por questões imediatas de sobrevivência, os participantes relataram a busca por serviços também para suporte às necessidades de higiene, refúgio e alimentação. Muitos priorizaram esse suporte em detrimento de outros cuidados ofertados.

*Sim, porque é uma questão de autocuidado* [...] *uma questão de, poxa, as questões básicas de higiene, alimentação.* (P3)

*Lá eles têm um trabalho, o Centro POP, eles dão um kitzinho, um tiquezinho para você almoçar, gratuito. É tipo um kit higiênico, que é um restaurante, sabonete, um creme dental, uma escova, um papel higiênico.* (P4)

*Fazer tratamento* [...] *poucos querem fazer tratamento* [...]. *Muitos querem ir só para comer e continuar na mesma vida.* (P7)

No que se refere à permanência nos serviços de atenção à saúde mental, os entrevistados destacaram o acesso contínuo e o acompanhamento nos serviços da RAPS, e que se organizam para retornarem às unidades e garantirem a continuidade por meio dos combinados realizados.

*Toda sexta-feira eu estou aqui. Fazendo tudo* [...]. *Passando no médico.* (P21)

*Então, o que a gente consegue minimamente se organizar, a gente vem procurando esse acesso e vem conseguindo, né?* (P3)

*Às vezes, ontem mesmo, eu estive no CAPS, amanhã eu vou estar lá de novo.* (P4)

*Na segunda, né? Vou voltar outras vezes, porque eles pediram, né, os que vão acompanhar melhor esse caso, né?* (P8)

Outros participantes relataram descontinuidade, seja por compromissos pessoais, pela percepção de que não necessitavam de assistência em saúde mental ou por vivências negativas, que geram resistência na busca por ajuda.

*Nesses dias eu não tô indo, porque tô resolvendo problemas. Trabalhando lá em Souzas, não tá dando pra ir. Faz o quê* [...] *uns três ou quatro meses que eu não vou.* (P13)

*Eu cheguei em uma época lá que eu pensei em abandonar o CAPS* [...] *por que eu quis trocar de CAPS? Porque eu achei que eu não estava tendo a atenção devida, sabe quando você sente, sei lá, desprezo.* (P4)

Entre as experiências negativas descritas, a PSR mencionou ter vivenciado situações de suporte inadequado, discriminação e preconceito, que ocasionaram percepções e sentimentos de exclusão, descaso e desconforto. Referiram ainda que, ao utilizarem serviços que indivíduos de outras realidades sociais acessam, como UBS e hospitais, o desconforto é acentuado. Para os entrevistados, esse cenário não se aplica ao CnaR, uma vez que reconheceram nesse serviço itinerante uma equipe que oferece tratamento diferenciado à PSR.

*Se você sofre um acidente, e está comigo ali, mesmo vivendo a vida que você vive, você vai ser tratado igual a mim. Você está sem documento, sem celular. ‘A hora que der, a gente vê’. É assim que funciona, ‘estou fechando’.* [...] *E é assim que é.* (P1)

*Existe alguma, não sei dizer, não posso dizer que é preconceito.* [...] *Mas existe um certo desconforto ao acessar esses serviços porque existem outras pessoas com outras realidades sociais acessando o mesmo serviço ao mesmo tempo. E a gente se sente um pouco desconfortável, mas talvez seja isso uma percepção só nossa também, né?* (P3)

*Só as pessoas* [...]. *Fazem desfeita da gente* [...]. *Só isso.* (P16)

*Pra pessoa te tratar diferente? Aqui* [CnaR]*. Porque os outros sistemas de saúde, não. Aqui eu não sei se são treinados, sei lá. Mas é um tratamento diferente, olho no olho, o que você está precisando. Escuta mesmo, conversa contigo, tudo mais.* (P1)

Essa estigmatização, segundo os entrevistados, se refletiu em julgamentos, aumento do tempo de espera para assistência e atendimentos insatisfatórios, além das vivências negativas se intensificarem quando usam vestimentas com sujidades e portam consigo pertences pessoais.

*Eu fiquei três horas sentado no banco, esperando pra ser atendido* [...] *chorando e rolando no chão. Pela situação de rua. Muita gente passou na minha frente, chegou e foi passando. E eu fui ficando pra trás. E eles julgam, né? Muita gente julga.* (P11)

*Porque eles falam “é morador de rua”, ninguém liga* [...] *“você mora na rua, você pode ficar horas e horas aí. A gente não vai atender agora”* [...]. *Outras pessoas que é bem vestida, que é da grana, rapidinho eles atendem* [...] *morador de rua, fica uma semana ali, eles não atendem direito, atendem mal.* (P13)

*Eu estava em outra situação, eu estava mal vestido, estava com cobertores, carregando essas coisas. Então, querendo ou não, até por uma questão cultural, isso acaba fazendo com que outras pessoas percebam de outra forma, né? A nossa realidade.* (P3)

Embora a PSR enfrente situações adversas e indesejáveis, alguns entrevistados enfatizaram experiências positivas nos serviços, como bons atendimentos. Também se referiram à possibilidade de redução do uso de drogas ao estabelecerem diálogos e relações com equipes da rede.

*Mas essa parte do atendimento do CnaR, o CAPS, os hospitais estão sendo bons. Eles atendem bem, tudo.* (P13)

*Pra mim, todos esses que eu fui são muito bons demais, viu?* [...] *Os médicos na rua, o SOS Rua. Pra mim, são ótimos.* (P20)

*O atendimento é maravilhoso. Carinho. Você vê que atendem a gente como gente.* (P21)

*Ah é, eles atendem a gente muito bem. São muito educados* [...]. *Principalmente o médico* [...]*. Quando o cara tá bruto, deitado lá, chapado, debaixo do pé do mato. Ele vai falar, se ajoelha perto da pessoa, conversa* [...]. *São ótimas pessoas.* (P15)

*Ela vem aqui só pra falar com alguém. Pra ouvir a voz de alguém, pra não ouvir falar que é ‘lixeira’, é sujo, é ‘nóia’* [...]. *Pra não usar droga ou pra tomar um gole de água, conversar com alguém. Respirar* [...]. *Porque isso aqui faz a gente se sentir gente.* (P1)

Quanto aos serviços disponíveis, depoimentos ilustraram a importância do CnaR para a assistência à saúde da PSR, visto que o atendimento é rápido, fluido, respeitoso e sem necessidade de agendamentos. Por fim, em razão de se tratar de um ambiente com objetivo de direcionar sua atenção à PSR, sentimentos de acolhimento são aludidos.

*Eu acho que faltaria bastante, entendeu? Se não tivesse esse trabalho aqui, eu acho que o povo que tá na rua ia perecer bastante.* (P10)

*Se não fosse hoje o CnaR, eu não conseguiria, talvez, uma receita, eu teria que agendar numa UBS, que demoraria dois meses um agendamento. Então, às vezes, a população de rua, inclusive, tem certos benefícios que a população em geral não tem. Porque uma consulta demora dois meses, eu chego aqui, eu sou atendido em dez minutos.* (P3)

*E aqui, o mais bacana do CnaR, tipo assim, é que ele trata o morador de rua igual qualquer outro* [...]. *Não tem aquela coisa assim* [...] *que ‘ai, você está fedendo porque você mora na rua’* [...]. *Entendeu? Maravilhoso.* (P21)

*O CnaR é um serviço, assim* [...] *é um serviço que tem um objetivo, esse objetivo é cumprido, sabe? E eles são muito carinhosos, muito atenciosos. E realmente fazem toda a diferença, esse serviço aqui.* (P3)

### Expectativas da população em situação de rua diante das experiências vividas

A experiência da PSR no acesso aos serviços da rede é complexa e multifacetada, o que gera expectativas que refletem seus anseios e os desafios vivenciados. Diante disso, o grupo trouxe que espera por uma assistência igualitária e universal, que garanta a todos o direito aos serviços de saúde. Portanto, a PSR relatou o desejo de que os profissionais da área da saúde atendam a todos, com uniformidade de tratamento, independentemente das características sociais ou pessoais.

*Mas isso não tem que ser diferente. Porque Sistema Único de Saúde é Sistema Único de Saúde. É para todo mundo.* (P1)

*Eu esperava que fosse uma questão de igualdade, somos cidadãos da mesma forma. Temos CPF igual outras pessoas, sangramos da mesma cor, entendeu? Então o sangue é vermelho* [...]. *Naquele dia, eu esperava ser tratado como uma pessoa, entre aspas, normal, porque todos nós somos normais, cada um com suas características, né?* (P3)

*A gente não tem que escolher quem a gente atende, não é verdade?* (P1)

*Porque eles tratam uma pessoa de um jeito, outra pessoa de outro jeito. Outra pessoa trata mal, outra pessoa trata bem* [...]. (P13)

Os participantes também se referiram ao desejo de serem reconhecidos como pessoas e não como estatística ou número de atendimentos. Anseiam por acolhimento, empatia e humanização para que as necessidades específicas do grupo sejam consideradas.

*Não quero ser estatística, não. Eu quero ser acolhido, eu quero ser ouvido.* (P1)

*Se a pessoa não for humana, não vai dar atenção pra outra, né? Porque hoje em dia as pessoas estão mais pensando em si próprio, né? Não pensando no próximo.* (P11)

A PSR mencionou a necessidade de atendimentos ágeis e o anseio de terem acesso a unidades e leitos de acolhimento, reconhecendo o papel desses na transição entre a rua e o retorno à sociedade. Para os entrevistados, o acesso a abrigos possibilitou a organização de pensamentos e de questões financeiras e sociais, fatores esses primordiais para a reinserção social e para o resgate da dignidade.

*Tem que ter uma coisa um pouco mais ágil pra poder resolver essa situação e às vezes olhar um pouco mais sobre a situação da mentalidade da pessoa que tá ali, passando aquela situação.* (P10)

*Eu não tô na rua porque eu quero. Tô na rua porque estou passando por dificuldade. Se pudesse me arrumar, ao menos um quarto, já era. Né? Mudaria isso.* (P14)

*É um outro serviço também* [Unidade de Acolhimento]*, que não sei se faz parte da rede das RAPS, mas é um serviço que vai me ajudar bastante nessa transição entre a situação de rua e a readaptação, o retorno à sociedade. Então, eu vou precisar desse período para me estabilizar, para eu conseguir isso do acolhimento, para eu poder me organizar, sabe? Organizar pensamentos, sentimentos, questões financeiras, sociais, até eu conseguir uma estabilidade financeira, principalmente social, para poder ter uma autonomia.* (P3)

*Eu acho que é o resgate da dignidade, né?* (P3)

Em contraponto, a PSR apontou ter vivenciado a assistência da maneira que esperava, ao ser cuidada com respeito e hospitalidade, de forma a corresponder às suas expectativas.

*Eu esperava, na minha mente, que seria desse jeito. Bem tratado, tratado como ser humano. Com respeito, com educação. Bem-vindo. Você se sente acolhido por eles.* (P7)

*Eu não acho que tem que mudar nada. Perfeito. Eu acho que tinha que ter mais. Porque aqui é melhor do que* [...] *tipo em atendimento, sabe? É um atendimento maravilhoso. Eu acho. Não tenho o que reclamar de nada. Nem da equipe, nada, sabe?* (P21)

## DISCUSSÃO

A aproximação com o mundo-vida da PSR possibilitou a compreensão de seu acesso aos serviços da RAPS, permitindo apreender as intencionalidades e as expectativas que orientam essa população na busca por cuidado na rede, fundamentadas em suas experiências vividas^(16)^. Diante disso, apesar da presença de diversas barreiras, observa-se que a PSR participante do estudo transita entre os pontos de atenção disponíveis na RAPS do município. A esse respeito, mencionaram o acesso aos serviços de atenção psicossocial, de atenção básica, assim como a serviços de urgência e emergência, hospitalares e de atenção residencial de caráter transitório. Tal achado contrasta com a literatura que indica que a PSR geralmente utiliza apenas serviços de urgência e emergência em situações de agudização^(13,14,24)^.

As experiências de acesso relatadas pela PSR participante do estudo ocorreram devido à estrutura da RAPS do município em que foi realizada a pesquisa, visto que detém atendimento e acolhimento do CnaR com equipes que apreendem as particularidades da PSR e a dinâmica do território em que circulam. Ressalta-se que o CnaR amplia a acessibilidade dessa população aos serviços de saúde ao articular demandas e ao promover o cuidado integral, enquanto sua ausência reduz a mobilidade da PSR, especialmente em áreas sem cobertura de atenção básica ou das equipes do CnaR^(13,19)^. Como contribuição deste estudo, destaca-se que a ausência de serviços de saúde compromete a mobilidade, o atendimento e o acesso da PSR à rede.

Entre as estratégias de acesso da PSR aos serviços da RAPS, depoimentos descreveram o contato com a rede decorrente de interações nas ruas, restaurantes populares, praças, abrigos e outros locais. Nesses espaços, a PSR difunde seus saberes e indica entre si locais para o cuidado à saúde mental nos quais previamente vivenciaram boas experiências. Esse dado revela a importância de estratégias de acolhimento adaptadas à PSR realizadas em espaços públicos em que os profissionais, por meio de interações informais^(25)^, estabelecem relações de confiança e empatia, o que favorece o vínculo e reduz barreiras de acesso ao cuidado em saúde mental^(26)^. Tais estratégias são guiadas pelo acervo de conhecimento e de experiências da PSR construído ao longo de suas trajetórias e por relações face a face caracterizadas como encontros autênticos em que sujeitos compartilham tempo e espaço, tornando-se mutuamente conscientes^(16)^.

O compartilhamento de vivências e as indicações de locais de cuidado permitem que a PSR busque serviços de assistência de forma mais autônoma, enquanto equipes como SOS Rua, CnaR e Centro POP, por meio de ações integradas, facilitam sua mobilidade e garantem encaminhamentos na rede. Essa evidência está em consonância com a literatura que ressalta a importância da acessibilidade da PSR aos serviços de saúde e a instituições socioassistenciais, com ações de apoio matricial, equipes especializadas^(13)^, horários de atendimento mais flexíveis e olhar atento para que a PSR não seja negligenciada^(27)^. A atuação dessas equipes, com abordagens estratégicas no mundo-vida cotidiano da PSR, e por meio da relação face a face no cuidar, pode minimizar barreiras de acesso, acolher e auxiliar essa população no reconhecimento de suas demandas^(13,16)^.

De acordo com os entrevistados, a busca por atenção na rede é motivada por implicações da vida nas ruas. A literatura aponta que a PSR busca principalmente serviços de urgência e emergência frente às demandas decorrentes de sua realidade, em que associam saúde à ausência de doenças e à capacidade funcional^(14,28)^. No entanto, também foi possível identificar um aumento da procura pela atenção primária, o que pode ser resultado do elevado número de doenças crônicas e do envelhecimento da PSR, sendo necessário assegurar o acesso à promoção e prevenção em saúde^(29)^. Além disso, a PSR disse acessar a rede para atendimentos com assistentes sociais, por desejo de transformação de seu mundo-vida, assim como para utilização de *internet*. O uso de tecnologia para promoção de saúde para essa população reforça que a conectividade proporciona comunicação com amigos e familiares, destacando sua influência na saúde mental^(10,30,31)^.

A PSR entrevistada apontou que, em muitos momentos, prioriza o cuidado às necessidades imediatas, como higiene e segurança alimentar, em detrimento dos demais cuidados ofertados na RAPS. Esse cenário decorre das distintas dimensões da vulnerabilidade vividas por essa população^(8)^. Há evidências na literatura que reforçam a necessidade de intersetorialidade^(32)^ e de abordagens colaborativas^(33)^ no campo da saúde mental visando o aprimoramento de ações para o cuidado integral, assim como para a compreensão das singularidades da PSR^(32,34)^.

Os achados ilustram o contraste entre a permanência e a descontinuidade nos serviços, estando essa interrupção associada a compromissos pessoais, à percepção de que não necessitam de cuidado em saúde mental e às vivências negativas na RAPS. O abandono do tratamento ocorre tanto em países de alta (30%) quanto de baixa renda (45%)^(35)^, estando associado à complexidades do sistema de saúde, a experiências negativas de acesso e à falta de confiança nos profissionais^(25)^.

Entre as adversidades ao acessar serviços da RAPS, a PSR citou situações de suporte inadequado, preconceitos e discriminações, o que ocasiona sentimentos de exclusão e desconforto. Mencionaram ainda que a sensação de desconforto é acentuada em serviços nos quais indivíduos de outras realidades sociais frequentam. Dessa forma, aponta-se que os serviços de saúde não estão preparados para receber e acolher as singularidades da PSR, razão pela qual o cuidado a grupos em vulnerabilidade se mostra como um constante desafio^(13,28,36)^. Além disso, a acessibilidade aos serviços de saúde mental pode ser comprometida por práticas profissionais influenciadas por estereótipos e julgamentos negativos^(13)^.

As vivências negativas são acentuadas quando a PSR frequenta a rede com pertences pessoais e asseio prejudicado, devido ao acesso precário a condições básicas de higiene^(13,14,28)^. Na perspectiva fenomenológica, os pertences da PSR podem refletir significados únicos de sua biografia de vida, como o cobertor, que simboliza abrigo físico e emocional^(16)^. Vivências negativas comprometem o cuidado e consolidam-se como entraves de acesso, contrariando os princípios de universalidade e equidade do SUS bem como os objetivos da PNPSR^(7,13,14)^.

Como experiência indesejável, a PSR também se referiu ao tempo de espera prolongado para assistência que, em sua perspectiva, está vinculado à estigmatização. Foi apontado que a permanência prolongada da PSR em unidades de atendimento ocasiona ansiedade, visto que eles se preocupam com suas atividades relacionadas à garantia da subsistência^(15)^. Assim, nota-se que o cuidado humanizado à PSR ainda é ofuscado por barreiras persistentes, marcadas pelo preconceito e estigma presentes em outros serviços da RAPS^(13)^.

De acordo com os participantes, o acesso à rede pode auxiliar na redução do uso de substâncias psicoativas, uma vez que assegura um encontro para acolhimento e diálogo com profissionais em momentos de vulnerabilidade. Enfatiza-se que o uso de tecnologias leves é essencial no cuidado em saúde mental, ao favorecer vínculos e encontros autênticos que conectam subjetividades por meio da relação face a face^(13,16)^, como é o caso da estratégia de “suporte de pares”, utilizada na redução de danos e conduzida por pessoas com vivências similares à PSR^(37)^.

Também foram ressaltadas experiências positivas, especialmente no CnaR. Os participantes apontaram a importância de o município dispor de CnaR para assistência à saúde mental, uma vez que esse dispõe de atendimentos rápidos, fluidos e sem a necessidade de agendamentos para consultas ou exames. Disseram ainda que nesses locais não se sentem excluídos pela equipe. Essa forma de atendimento é relevante para a PSR, uma vez que a exigência de horários marcados e a espera em filas podem dificultar o acesso à sua alimentação diária devido à sobreposição com horários de funcionamento de restaurantes populares, por exemplo^(38)^. Tendo em vista que a insegurança alimentar pode comprometer a continuidade de cuidados^(38)^, reforça-se a importância de serviços que conciliem acolhimento, flexibilidade e acessibilidade no cuidado em saúde mental, como ocorre no CnaR.

A PSR também exteriorizou diversas intencionalidades, como ter assistência igualitária e universal na qual os profissionais da saúde atendam todos com uniformidade e humanização. Buscam serem reconhecidos como pessoas e não estatística ou número de atendimentos. Portanto, é relevante a atuação de profissionais que adotem práticas acolhedoras e empáticas no cuidado à PSR de forma a contribuírem para a construção de percursos terapêuticos sensíveis às especificidades do processo saúde-doença^(39)^ ao reconhecerem o sujeito como ator social inserido em seu mundo-vida^(16)^.

Os serviços da RAPS ainda enfrentam desafios para assistir o usuário com equidade e empatia, dada a persistência de concepções estigmatizadas e estereotipadas, o que evidencia a necessidade de formar profissionais para atuar com sensibilidade frente às complexidades envolvidas no cuidado em saúde mental, bem como a urgência da promoção de processos contínuos de qualificação das equipes, por meio da educação permanente visando à construção de práticas inclusivas e transformadoras^(5)^. Esse perfil esperado pode ser visto em relatos dos entrevistados, ao mencionarem o anseio por atendimentos com empatia e humanização e pela consideração de suas singularidades.

Diante da experiência vivida na rede, outra expectativa da PSR é o acesso a unidades e leitos de acolhimento, ao destacar a importância de um abrigo para sua saúde e reinserção na sociedade, pois ter refúgio auxilia no processo de organização psicossocial e financeira e no resgate da dignidade. Essa relação pode ser observada no modelo de determinação social da saúde, que compreende a saúde como fenômeno socialmente construído, resultado de processo históricos e estruturais, como trabalho, relações de classe e políticas públicas^(40)^, os quais permeiam o mundo-vida do indivíduo e influenciam diretamente em sua saúde^(16)^. Desse modo, o déficit de espaços para abrigo temporário e as condições inadequadas de habitação influenciam no processo saúde-doença da PSR, enquanto a existência deles, por outro lado, promove a reinserção social e aumenta a chance de vínculo empregatício formal, essencial para essa reintegração^(11)^.

Por fim, muitos entrevistados relataram que o atendimento ocorre de modo esperado, com hospitalidade e respeito, correspondendo às suas expectativas. O cuidado em saúde, independentemente da pessoa atendida, deve considerar o contexto social e ser sustentado por profissionais que a reconheçam enquanto sujeito de direitos, aspecto indispensável na relação profissional-pessoa que recebe cuidados^(19)^. Esse olhar ampliado pode decorrer das relações intersubjetivas, nas quais a PSR é reconhecida e suas experiências, situação biográfica e acervo de conhecimento são valorizados^(16)^.

### Limitações do estudo

Quanto às limitações, enfatiza-se que a pesquisa esteve restrita a apenas um município, território com atuação do CnaR em que a delimitação territorial, as características locais e a oferta de serviços podem influenciar nas dinâmicas de acesso e cuidado observadas em outros cenários.

### Contribuições para a área

Como contribuição para a área de enfermagem, este estudo amplia a compreensão do acesso da PSR aos serviços da RAPS e oferece subsídios para que enfermeiros atuem de forma sensível, crítica e comprometida. Ao compreender as motivações e significados atribuídos às ações da PSR, o enfermeiro pode qualificar sua escuta, adaptar estratégias de cuidado e fortalecer vínculos terapêuticos. Assim, ao desvelar o fenômeno sob o olhar de quem o vivencia, espera-se que o estudo contribua para a visibilidade social da PSR e fortaleça sua voz na sociedade. Ademais, enfatiza-se a importância do CnaR, visto que esse serviço permite novas dinâmicas de acesso à rede para quem vivencia a rua em seu mundo-vida.

## CONSIDERAÇÕES FINAIS

Este estudo possibilitou compreender o acesso à RAPS na perspectiva da PSR em um município do interior paulista. Observou-se que esse acesso é permeado pelas experiências vividas nas ruas, sendo moldado pela vulnerabilidade e pela situação biográfica da PSR, aspectos que configuram os “motivos-porque”. Os “motivos-para” revelam as expectativas e os projetos de vida dos entrevistados, de maneira a indicar direções futuras desejadas dentro do seu percurso biográfico.

Ao unir e compreender os motivos existenciais dessa população, tem-se a construção do típico da ação: a PSR apresenta mobilidade significativa na rede, facilitada pela atuação do CnaR e pela articulação de equipes assistenciais. O acesso ocorre por meio de interações sociais em ambientes de convivência, onde conhecimentos sobre os serviços são compartilhados. Contudo, foi identificada uma dualidade entre permanência e descontinuidade no cuidado, vinculada às experiências vividas. A PSR destacou o acolhimento e bons atendimentos, mas também apontou discriminações e suporte inadequado em serviços que atendem realidades sociais diversas.

Entre as expectativas, ressaltou-se o desejo por assistência igualitária e universal, além de seu reconhecimento como indivíduo e não estatística. Para atender a essas demandas, reforça-se a necessidade de práticas acolhedoras, empáticas e sensíveis por parte dos profissionais de saúde, alinhadas aos princípios do SUS e da PNPSR. Diante das barreiras e do estigma enfrentados, sugere-se a realização de novos estudos, com foco na atuação profissional e no fortalecimento e qualificação do acesso à rede.

## Data Availability

Os dados de pesquisa estão disponíveis no corpo do artigo.
